# *Pseudomonadota* bridge cross-trophic interactions to suppress plant pathogens

**DOI:** 10.1093/ismejo/wrag011

**Published:** 2026-01-28

**Authors:** Huiyu Chuai, Gen Li, Luchen Tao, Lei Ouyang, Ruihan Ruan, Zhong Wei, Joann Whalen, Uffe N Nielsen, Ting Liu, Huixin Li

**Affiliations:** Asia Hub on Agriculture, Sanya Institute of Nanjing Agricultural University, Sanya, Hainan 572024, China; Soil Ecology Lab, College of Resources and Environmental Sciences, Nanjing Agricultural University, Nanjing, Jiangsu 210095, China; Jiangsu Collaborative Innovation Center for Solid Organic Waste Resource Utilization, Nanjing Agricultural University, Nanjing, Jiangsu 210095, China; Asia Hub on Agriculture, Sanya Institute of Nanjing Agricultural University, Sanya, Hainan 572024, China; Soil Ecology Lab, College of Resources and Environmental Sciences, Nanjing Agricultural University, Nanjing, Jiangsu 210095, China; Jiangsu Collaborative Innovation Center for Solid Organic Waste Resource Utilization, Nanjing Agricultural University, Nanjing, Jiangsu 210095, China; Asia Hub on Agriculture, Sanya Institute of Nanjing Agricultural University, Sanya, Hainan 572024, China; Soil Ecology Lab, College of Resources and Environmental Sciences, Nanjing Agricultural University, Nanjing, Jiangsu 210095, China; Asia Hub on Agriculture, Sanya Institute of Nanjing Agricultural University, Sanya, Hainan 572024, China; Soil Ecology Lab, College of Resources and Environmental Sciences, Nanjing Agricultural University, Nanjing, Jiangsu 210095, China; Asia Hub on Agriculture, Sanya Institute of Nanjing Agricultural University, Sanya, Hainan 572024, China; Soil Ecology Lab, College of Resources and Environmental Sciences, Nanjing Agricultural University, Nanjing, Jiangsu 210095, China; Jiangsu Collaborative Innovation Center for Solid Organic Waste Resource Utilization, Nanjing Agricultural University, Nanjing, Jiangsu 210095, China; Department of Natural Resource Sciences, McGill University, Montreal, QC H9X 3V9, Canada; Chair of Soil Science, Mohammed VI Polytechnic University, Ben Guerir, Marrakech-Safi 43150, Morocco; Hawkesbury Institute for the Environment, Western Sydney University, Penrith, NSW 2751, Australia; Asia Hub on Agriculture, Sanya Institute of Nanjing Agricultural University, Sanya, Hainan 572024, China; Soil Ecology Lab, College of Resources and Environmental Sciences, Nanjing Agricultural University, Nanjing, Jiangsu 210095, China; Asia Hub on Agriculture, Sanya Institute of Nanjing Agricultural University, Sanya, Hainan 572024, China; Soil Ecology Lab, College of Resources and Environmental Sciences, Nanjing Agricultural University, Nanjing, Jiangsu 210095, China

**Keywords:** soil nematodes, predator–prey interactions, bacterial traits, plant pathogens

## Abstract

Trait-mediated interactions across trophic levels drive trophic cascades in macroecological systems, yet their relevance in microbially dominated soil ecosystems remains underexplored. We combined a regional field survey with controlled experiments using a defined 122-strain synthetic bacterial community and bacterivorous nematodes to test whether faunal predation reorganizes root-associated microbiomes to suppress soilborne disease. Field observations showed that sites with stronger nematode-*Pseudomonadota* associations had lower bacterial wilt incidence. In controlled experiments, nematode predation selectively enriched *Pseudomonadota* in the rhizosphere and reduced *Ralstonia solanacearum* populations and disease incidence. Preferential grazing drove this enrichment: *Pseudomonadota* constituted over 95% of sequences in nematode guts, and focal taxa showed moderate antagonism, small cell size, and high metabolic activity. Together, these results identify *Pseudomonadota* as key bridging taxa in cross-trophic interactions. Trait-linked responses to predation contribute to pathogen suppression and suggest a biocontrol framework that integrates microbial traits with trophic connectivity.

## Introduction

Trait-mediated interactions across trophic levels are recognized as key drivers of community structure, ecosystem functioning, and evolutionary dynamics [1, 2]. These interactions arise when consumers filter prey based on functional traits, thereby altering the abundance, composition, or ecological roles of lower trophic groups [3, 4]. Whereas this mechanism is well established in macroecosystems [5], its prevalence and ecological consequences in microbially dominated soils remain poorly understood [6]. Yet, cross-trophic interactions in soil microbial systems play fundamental roles in maintaining biodiversity and stabilizing population dynamics [7, 8]. Deciphering their underlying mechanisms is challenging due to the microscopic scale, high taxonomic diversity, and rapid turnover of soil microbiota [6, 9]. These difficulties are further compounded by predator feeding complexity and technical limitations in resolving predator–prey links, which are more easily identified in macroscopic systems using gut content analysis [10–12]. As a result, microbial ecology has historically focused on within-trophic interactions such as competition and cooperation [13–15], whereas the functional roles of trait-mediated cross-trophic interactions remain largely unexplored [16–18].

This study explains how cross-trophic interactions contribute to plant pathogen suppression, with evidence spanning from large-scale field investigations to controlled experiments using synthetic microbial communities. Nematodes were selected as model microbial predators [19, 20], and *Ralstonia solanacearum*, a globally distributed soilborne bacterium that causes bacterial wilt in over 200 plant species [21, 22], served as the model pathogen. Our first objective was to identify key microbial players in cross-trophic interactions affecting *R. solanacearum* populations. To achieve this, we analyzed micro-food web structures across 100 field sites with and without *R. solanacearum* infection and constructed a synthetic microbial community comprising dominant bacterial phyla known for their pathogen-suppressive ability: *Actinobacteria* (e.g. *Arthrobacter*) [17], *Firmicutes* (e.g. *Bacillus*) [23, 24], *Pseudomonadota* (e.g. *Pseudomonas*) [25, 26], and *Bacteroidetes* (e.g. *Flavobacterium*) [27, 28].

Our second objective was to examine how nematode predation influences these bacterial groups, focusing on selective feeding behavior that may reflect trait-based ecological filtering. Nematode predation often creates positive feedback loops that enhance bacterial activity and function [20, 29], though its impact on bacterial biomass can vary with grazing intensity and may increase or decrease under experimental conditions [30, 31]. Like macropredators, nematodes exhibit selective feeding behavior—favoring *Alphaproteobacteria* [32–35] whereas avoiding Gram-positive bacteria with thick cell walls [19, 36] or toxic species that impair their growth [6, 37–39]—with these preferences potentially shaped by prey traits such as cell wall structure and metabolite production. Such trait-driven feeding may bias bacterial community assembly toward *Pseudomonadota*, establishing cross-trophic links between predator behavior and downstream microbial functions in pathogen suppression.

**Figure 1:**
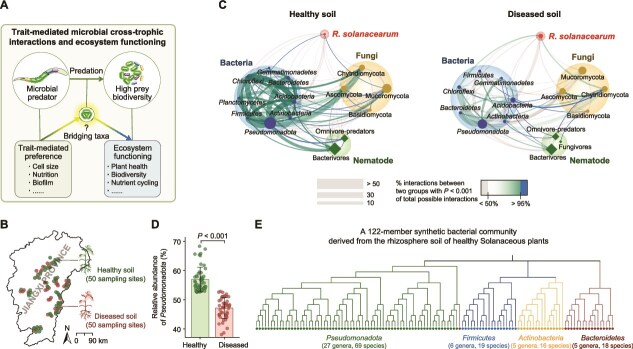
Cross-trophic interactions mediated by *Pseudomonadota* and bacterivores modulate bacterial wilt disease in plants. (A) Conceptual framework highlighting how microbial predators, via trait-mediated prey selection, interact with prey diversity to influence ecosystem function. Bridging taxa act as key mediators, connecting predation dynamics to ecosystem outcomes such as plant health and biodiversity. (B) Regional map showing the locations of 50 sampling sites with rhizosphere soils from healthy plants and 50 sites with *R. solanacearum*–infected soils from diseased plants across Jiangxi Province, China. (C) Healthy soils show stronger interactions among *Pseudomonadota* and bacterivores compared to diseased soils (*n* = 50 per treatment). Co-occurrence networks were constructed using SparCC (|*r*| > 0.3, *P* < .05), focusing on nodes representing the top 95% of total relative abundance and emphasizing connections involving *R. solanacearum* and nematodes. Line width indicates the number of correlations, while line color and transparency reflect interaction strength. The size of circles and squares corresponds to the relative abundance of taxa. Interaction strength was calculated by dividing the number of highly significant correlations by the total possible interactions between two groups. (D) Healthy soils have a greater relative abundance of *Pseudomonadota* compared to diseased soils (*n* = 50 per treatment). (E) Phylogenetic tree of 122 bacterial strains isolated and cultured from healthy rhizosphere soils and selected as representative dominant taxa for subsequent pot experiments.

To achieve these objectives, we established a microbial predator–prey system using 122 bacterial strains representing dominant cultivable taxa in the Solanaceous plant rhizosphere, including *Pseudomonadota*, *Bacteroidetes*, *Firmicutes*, and *Actinobacteria*. These groups display distinct functional traits increasingly recognized as drivers of soil food web structure and ecosystem processes [6, 40]. We hypothesize that *Pseudomonadota* serve as “bridging taxa,” mediating cross-trophic interactions through trait-mediated selection and linking nematode predation to downstream effects on pathogen suppression (Fig. 1A). To test this, we sequenced the genomes of the strains and experimentally quantified traits relevant to predator–prey interactions—including cell size, Gram identity, metabolic activity, protein and carbohydrate content, and antagonistic ability [6, 19]. By tracking bacterial enrichment in both the rhizosphere and nematode guts, we assessed how these traits influence nematode feeding preferences and their role in pathogen suppression.

## Materials and methods

### Sample collection and DNA extraction

We collected rhizosphere soil from 100 Solanaceous plant sites in Jiangxi Province, China—50 from wilted plants infected by *R. solanacearum* (diseased soils) and 50 from asymptomatic plants (healthy soils). The distance between healthy and diseased sites ranged from several meters to a few kilometers, depending on field layout and disease distribution. At each site, rhizosphere soils from 8 to 10 plants spaced at least 1 m apart were collected and combined into a single composite plot sample. In the field, roots with adhering soil were carefully excavated, and loosely attached bulk soil was gently removed by shaking. Samples were transported to the laboratory on dry ice. Rhizosphere soil was defined as the soil tightly adhering to the root surface and was collected under standardized laboratory conditions by suspending the roots in sterile phosphate-buffered saline (PBS), followed by centrifugation at 10 000 × g for 10 min. DNA was extracted from 0.5 g of rhizosphere soil from each sample using the E.Z.N.A. Soil DNA Kit (Omega Bio-tek, Norcross, GA, U.S.), according to the manufacturer’s instructions. Extracted DNA was used for characterizing bacterial, fungal, and nematode communities.

### Polymerase chain reaction (PCR) amplification and high-throughput sequencing

The V4–V5 region of the bacterial 16S ribosomal RNA (rRNA) gene and the V4 region of the fungal and nematode 18S rRNA gene were amplified using the primer sets 515F/907R [41] and 528F/706R [42], respectively, according to previously established methods. Taxonomic classification of each amplicon sequence variant (ASV) was performed using the lowest common ancestor (LDA) algorithm against the SILVA SSU138.1 16S rRNA database for bacteria, the NCBI nucleotide (NT)-fungi database for fungi, and the SILVA-nematode database for nematodes. PCR amplification was performed in a 20 μl reaction volume containing 4 μl of 5× FastPfu Buffer, 2 μl of deoxynucleoside triphosphates (dNTPs; 2.5 mM), 0.8 μl of each primer (5 μM), 0.4 μl of FastPfu polymerase, and 10 ng of DNA. The amplification conditions were: an initial denaturation at 95°C for 2 min, 25 cycles of denaturation at 95°C for 30 s, annealing at 55°C for 30 s, extension at 72°C for 45 s, and a final extension at 72°C for 10 min. PCR products were pooled at equimolar concentrations (10 ng μl^−1^) and sequenced using paired-end technology on a MiSeq System (Illumina) at Shanghai BIOZERON Co., Ltd (Shanghai, China).

### Isolation and identification of 122 bacterial strains

The 122 bacterial strains used to construct the synthetic community (SynCom) were obtained from a long-term culture collection built over several years from the rhizosphere soils of healthy Solanaceous plants (e.g. tomato, tobacco) across several provinces in South China. A subset of isolates also originated from the agricultural fields sampled in this study. Isolates were selected because they were recurrent and relatively abundant across sites, culturable, and showed *in vitro* antagonism against *R. solanacearum*. Briefly, soil samples were resuspended in sterile 0.9% NaCl, and aliquots were plated onto Tryptic Soy Broth (TSB) medium (15 g tryptone, 5 g soytone, 2.5 g glucose, and 5 g NaCl per liter of deionized water) and/or mannitol agar plates. The plates were incubated at 20°C for 1–2 days. Single colonies were then isolated and cultured overnight in the same medium with shaking at 200 rpm at 28°C or until significant growth was observed. The resulting bacterial cultures were preserved in 15% (v/v) glycerol stocks and stored at −80°C. Bacterial isolates were identified by amplifying and sequencing a fragment of the 16S rRNA genes (Table S1).

### Isolation and cultivation of two nematode species

Two bacterivorous nematodes, *Panagrolaimus* sp. and *Distolabrellus veechi*, were isolated from the rhizosphere of tomato plants using a modified Baermann funnel method [43]. Nematodes were synchronized for three to four generations by bleaching, which lyses adults and sterilizes surface microbes while retaining eggs that carry minimal bacteria, thereby reducing and homogenizing background bacteria. Their identities were confirmed by morphological characteristics and 18S rRNA gene sequencing. Both species were maintained at 20°C on nematode growth medium (NGM) seeded with *Escherichia coli* OP50. NGM was prepared by mixing 3 g NaCl, 2.5 g peptone, 17 g agar, and 975 ml H₂O, followed by autoclaving. After cooling to 55°C, the medium was supplemented with 25 ml of 1 M KPO₄ buffer, 1 ml of 1 M CaCl₂, 1 ml of 1 M MgSO₄, and 1 ml of 5 mg ml^−1^ cholesterol in ethanol. The medium was adjusted to pH 6.0 and filtered through a 0.22 μm membrane before use.

### Pot experiment examining cross-trophic interactions in the suppression of *R. solanacearum*

The 122 bacterial strains were individually cultured in fresh TSB at 28°C with shaking at 200 rpm until the OD_600_ reached 0.6. Cultures were then combined in equal volumes to form SynCom. *Ralstonia solanacearum* was cultured in fresh Nutrient Broth (NB) medium (10 g glucose, 3 g beef extract, 5 g peptone, and 0.5 g yeast extract in 1 l deionized water) at 28°C with shaking at 200 rpm until the OD_600_ reached 0.6. All bacterial cultures were washed three times with sterile 10 mM PBS and resuspended in sterile water to a final density of 1 × 10^6^ cells ml^−1^. The *Panagrolaimus* sp. and *D*. *veechi* nematode species were individually cultured on NGM at 20°C for three to four generations. Nematodes were then washed three times with sterile 10 mM PBS and resuspended in sterile water to a final density of 1 × 10^3^ individuals ml^−1^. All inoculations were performed using fresh cultures.

The soil used in the pot experiment was a yellow-brown soil, classified as a Luvisol under the Food and Agriculture Organization (FAO) system. Its basic physicochemical properties were pH 6.07, soil organic carbon 16.4 g kg^−1^, total nitrogen 1.5 g kg^−1^, and total phosphorus 0.3 g kg^−1^. Before use, the soil was homogenized, mixed 1:1 (v/v) with a commercial nursery substrate and sterilized by gamma irradiation to eliminate indigenous microorganisms.

**Figure 2:**
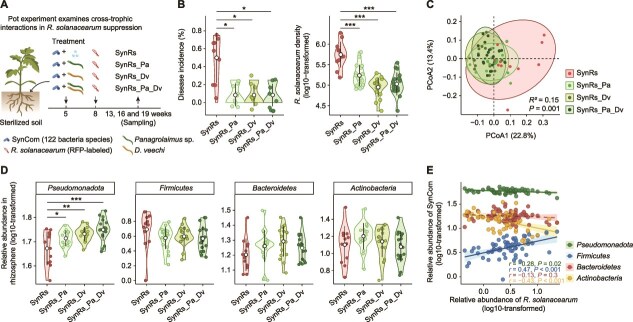
Bacterivore predation enriches *Pseudomonadota* and reduces plant disease. (A) Experimental design assessing cross-trophic interactions between bacterivores and bacteria and their effects on *R. solanacearum* invasion. The SynRs treatment involves inoculating a synthetic community of 122 bacterial species (SynCom) along with red fluorescent protein (RFP)-labeled *R. solanacearum* into tomato plant roots. Cross-trophic treatments include the SynCom, RFP-labeled *R. solanacearum*, and bacterivorous nematodes — *Panagrolaimus* sp. (SynRs_Pa), *Distolabrellus veechi* (SynRs_Dv), or both (SynRs_Pa_Dv)—inoculated into tomato plants (*n* = 18 pots/plants per treatment). (B) Nematode inoculation reduces disease incidence and the density of *R. solanacearum* in the rhizosphere. Significant differences marked with “*,” “**,” and “***” indicate *P* < .05, *P* < .01, and *P* < .001, respectively. (C) Nematode inoculation changes bacterial community composition (*n* = 18 across three sampling times per treatment). (D) Nematode inoculation enriches *Pseudomonadota* in the rhizosphere (*n* = 18 across three sampling times per treatment). (E) Relative abundance of rhizosphere *Pseudomonadota* is negatively correlated with the abundance of *R. solanacearum* (*n* = 72 across three sampling times and four treatments). Shaded areas represent 95% confidence intervals.

The pot experiment involved growing tomato plants under four treatments: (i) SynRs: SynCom (1.0 × 10^5^ cells g^−1^ dry soil) and *R. solanacearum* (1.0 × 10^4^ cells g^−1^ dry soil) were inoculated. (ii) SynRs_Pa: SynCom (1.0 × 10^5^ cells g^−1^ dry soil), nematode species *Panagrolaimus* sp. (10 individuals g^−1^ dry soil), and *R. solanacearum* (1.0 × 10^4^ cells g^−1^ dry soil) were inoculated. (iii) SynRs_Dv: SynCom (1.0 × 10^5^ cells g^−1^ dry soil), nematode species *D. veechi* (10 individuals g^−1^ dry soil), and *R. solanacearum* (1.0 × 10^4^ cells g^−1^ dry soil) were inoculated. (iv) SynRs_Pa_Dv: SynCom (1.0 × 10^5^ cells g^−1^ dry soil), *Panagrolaimus* sp. (5 individuals g^−1^ dry soil), *D*. *veechi* (5 individuals g^−1^ dry soil), and *R. solanacearum* (1.0 × 10^4^ cells g^−1^ dry soil) were inoculated (Fig. 2A). Each treatment was replicated six times across three sampling time points (18 replicates in total).

In brief, surface-sterilized tomato seeds were sown in sterile soil (100 g dry soil per pot; 6 cm × 6 cm × 10 cm pots) and irrigated with sterile water. At 5 weeks of growth, SynCom and the two nematode species were introduced into the relevant pots as per the treatment design. *Ralstonia solanacearum* was subsequently inoculated at 8 weeks. Plant disease symptoms were monitored, and destructive sampling was conducted at Weeks 13, 16, and 19. At each sampling point, both the rhizosphere and nematode gut samples were collected to quantify the abundance of SynCom members and *R. solanacearum* by 16S rRNA gene sequencing on the MiSeq System (Illumina), following the LorMe synthetic community high-throughput sequencing and bioinformatics analysis guidelines (http://yaozhongs-organization.gitbook.io/lorme-csc-database/). Rhizosphere samples were collected as previously described; nematode gut samples were obtained by extracting nematodes from 100 g of soil by centrifugation-flotation and counting their numbers, surface-sterilized with 2% NaOH and 0.2% NaClO, and then crushed to obtain gut contents.

### Quantification of *R. solanacearum* in the rhizosphere

Total genomic DNA was extracted from rhizosphere soil samples following previously established protocols. The abundance of *R. solanacearum* was quantified using specific primers (forward: 5′-AGTTCATGTACGGCTCCAAGGCCTAC-3′; reverse: 5′-CGCGCAGCTTCACCTTGTAGATGAAC-3′). A standard curve was generated based on established methods to ensure accurate quantification. Quantitative PCR (qPCR) assays were performed on a QuantStudio 3 Real-Time PCR System (Thermo Fisher Scientific, Waltham, MA, USA). Each sample was analyzed in three technical replicates, and the abundance was expressed as log₁₀-transformed gene copies g^−1^ soil.

### Nematode grazing intensity under mild *R. solanacearum* preconditioning

A suspension of *R. solanacearum* was evenly spread onto fresh NGM and incubated at 30°C for 12 h. After incubation, ~500 L4-stage individuals of *Panagrolaimus* sp. or *D*. *veechi* were separately introduced onto each plate. The plates were then immediately transferred to a dark incubator at 20°C for 6 h. This mild preconditioning was used to mimic realistic rhizosphere exposure to the pathogen while avoiding acute stress that could confound subsequent feeding responses, thereby standardizing nematode physiological state prior to grazing assays. Nematodes were collected and washed three times with sterile 10 mM PBS, starved for 12 h in M9 buffer, and then washed an additional three times to ensure minimal residual nutrients and bacteria.

To evaluate the effects of bacterial strains on nematode grazing, 29 bacterial strains were individually inoculated into fresh TSB and cultured at 28°C with shaking at 200 rpm until the OD_600_ reached 0.6. For each treatment, one bacterial suspension and ~500 individuals of one nematode species preconditioned under mild *R. solanacearum* stress were transferred to a 12-well plate. The total volume was adjusted to 1.5 ml using a cultivation solution of 10 mM PBS containing 6% TSB. Wells without nematodes served as controls, and each treatment was replicated six times across two nematode species (12 replicates in total). The plates were then immediately placed in a dark shaker at 20°C and shaken at 120 rpm for 72 h. After incubation, the OD_600_ values were measured to assess bacterial density. Nematode grazing intensity was calculated as OD*c–*OD*t*, where OD*c* and OD*t* represent the OD_600_ values of the control (without nematodes) and the treatment (with nematodes), respectively.

### Bacterial antagonistic ability against *R. solanacearum*

The 29 bacterial strains were individually inoculated into fresh TSB and cultured at 28°C with shaking at 200 rpm until OD_600_ reached 1. After incubation, cultures were filtered through sterile 0.22 μm membranes to obtain extracellular secretions. Each extracellular secretion was then used to culture *R. solanacearum*, with fresh TSB serving as the control. Each treatment was replicated eight times. After 24 h of incubation, OD_600_ values were measured to assess bacterial density. The antagonistic ability of each bacterial strain was calculated as OD*c*–OD*t*, where OD*c* is the OD_600_ of the control (cultured in fresh TSB), and OD*t* is the OD_600_ of the treatment (cultured with bacterial extracellular secretions).

### Assessment of bacterial traits

We evaluated six key traits for each of the 29 bacterial strains:

(1) Cell size: Each bacterium cultured to the stationary phase was washed with sterile saline solution and transferred to a glass slide. Cells were stained with crystal violet, and their width and length were measured using a microscope equipped with Motic Images Plus 3.0 software.

(2) Gram [44]: Each bacterial culture was washed with sterile saline solution and transferred to a glass slide for Gram staining to determine whether the bacteria were Gram-positive or -negative.

(3) Total protein content and (4) total carbohydrate content [45]: Washed bacteria were adjusted to an OD_600_ of 0.5 and sonicated with a VCX600 (Sonics, USA). The supernatant was then divided into two equal portions following centrifugation at 15 000 × g for 10 min to remove cell debris. One portion was analyzed for total protein using the Coomassie Brilliant Blue method with the G250 Protein Kit (mlBIO, China), whereas the other was assessed for total carbohydrate content using the phenol-sulfuric acid method. Each treatment was replicated three times.

(5) Activity [46]: A 25 ml bacterial suspension (adjusted to OD_600_ = 0.5 in TSB) was added to a sealed bottle preflushed with CO₂-free air, resulting in a headspace CO₂ concentration of ~0 μg ml^−1^. The bottle was incubated at 20°C for 12 h, after which the CO₂ concentration in the headspace was measured using gas chromatography (Agilent 7890B, USA), equipped with a flame ionization detector. The oven and detector were set to 55°C and 230°C, respectively. Each treatment was replicated three times. The activity (1 U) was defined as 1 mg of CO_2_ produced by each bacterium per 12 h.

(6) Secondary metabolite prediction [47]: Secondary metabolite biosynthetic potential was assessed using antiSMASH version 7.1 (https://antismash.secondarymetabolites.org/#!/start). The genomic sequences of each bacterial strain were uploaded to the antiSMASH platform, and analyses were conducted using the *Known Cluster Blast*, *Active Site Finder*, *SubCluster Blast*, and *RREFinder* modes. These analyses systematically scanned the genomic data to identify biosynthetic gene clusters (BGCs) responsible for secondary metabolite production. Each BGC corresponds to a potential secondary metabolite, such as antibiotics, antifungals, or other bioactive compounds. Visualized outputs detailed the structures of gene clusters and gene annotations, with highlights indicating the associations of each gene cluster with secondary metabolites.

### Construction and analysis of co-occurrence network

Based on ASV datasets encompassing bacteria, fungi, and nematodes, bacterial and fungal ASVs were filtered using thresholds of relative abundance >0.01% and occurrence rate >10%. Three nematode functional groups—bacterivores, fungivores, and omnivore-predators—were selected to construct comprehensive symbiotic networks for healthy and diseased soil samples. Comprehensive symbiotic networks were constructed using the network inference tool SparCC [48, 49] on the Integrated Network Analysis Pipeline (iNAP) platform, with default significance thresholds of |*r*| > 0.3 and *P* < .05. In these networks, nodes represent ASVs assigned to genera, whereas edges represent significant correlations between ASVs. To explore the interactions involving *R. solanacearum* and nematodes, bacterial and fungal ASVs associated with these taxa were extracted from the comprehensive symbiotic networks to construct subnetworks. These subnetworks were further classified according to bacterial and fungal phyla and nematode functional groups, focusing on nodes representing the top 95% of the total relative abundance for in-depth analysis. These correlation networks were visualized using Cytoscape (version 3.10.1) and Gephi (version 0.10.1) software.

### Statistical analyses

All statistical analyses and visualizations were performed in R (version 4.1.2), with all figures generated using the *ggplot2* package except for the regional distribution map, which was created using ArcGIS (version 10.8).

To integrate phylogenetic relationships with bacterial ecological traits, a maximum-likelihood phylogenetic tree was constructed in MEGA (version 11) using aligned 16S rRNA gene sequences from representative bacterial taxa. Trait data, including grazing intensity, antagonistic ability, and the relative abundance of bacteria in nematode guts (scaled from 0 to 1), were overlaid onto the tree and visualized as a composite heat map using the Interactive Tree of Life platform (https://itol.embl.de/).

To infer microbial functional profiles from the regional survey, Kyoto Encyclopedia of Genes and Genomes (KEGG)-based metabolic pathway predictions were generated using PICRUSt2 (https://www.bioincloud.tech/standalone-task-ui/picrust2) based on 16S rRNA gene data. Pathways related to microbial antagonism were selected for downstream comparison.

To assess microbial community structure and diversity, principal coordinate analysis based on Bray–Curtis dissimilarity was used to evaluate differences in bacterial, fungal, and nematode communities between healthy and diseased soils, as well as among SynRs and cross-trophic treatments. Statistical significance in community composition was tested using permutational multivariate analysis of variance (PERMANOVA) via the adonis function. One-way ANOVA followed by Tukey’s honestly significant difference (HSD) *post hoc* test (for normally distributed data), or Kruskal–Wallis test followed by Dunn’s test (for non-normal data), was applied to compare the following variables across treatments: (i) relative abundances of bacterial and fungal phyla, and nematode functional groups; (ii) Shannon diversity and richness of bacterial, fungal, and nematode communities; (iii) disease incidence, pathogen density, and the abundances of major bacterial phyla (*Pseudomonadota*, *Firmicutes*, *Bacteroidetes*, and *Actinobacteria*); (iv) bacterial traits, nematode grazing intensity, and the antagonistic ability of bacterial phyla against *R. solanacearum*; (v) antagonistic ability among low, moderate, and high groups; and (vi) predicted abundances of bacterial functional pathways. Pearson correlation (for normally distributed data) or Spearman correlation (for non-normal data) was used to examine the following relationships: (i) abundances of major bacterial phyla and *R. solanacearum* in the rhizosphere; (ii) abundances of *Pseudomonadota* classes and *R. solanacearum* in the rhizosphere; and (iii) abundances of *Pseudomonadota* classes in nematode guts and *Pseudomonadota* phylum in the rhizosphere.

To identify microbial taxa and traits associated with pathogen suppression and nematode grazing preference, we fitted generalized linear mixed-effects models (GLMMs) using the glmer function from the *lme4* package. Two modeling scenarios were evaluated: (i) rhizosphere bacterial phyla predicting *R. solanacearum* abundance and (ii) bacterial traits predicting nematode grazing intensity. In all models, predictor variables were treated as fixed effects. Sampling time, replicate, or bacterial species identity (as appropriate) was included as a random effect to account for repeated measures. For grazing models, bacterial traits included cell size, Gram identity, protein content, carbohydrate content, activity, and BGC richness. In Model (ii), antagonistic ability was also included as a categorical variable (low, moderate, high) based on tertile classification. Prevalence ratios were calculated to assess effect sizes: values >1 indicate a positive association with the response variable (e.g. higher grazing intensity), whereas values <1 indicate a negative association. To estimate the relative importance of each predictor, we used the *glmm.hp* package, which partitions the variance explained by fixed effects within the mixed-effects framework [50, 51].

## Results

### Strong cross-trophic microbial interactions are associated with lower bacterial wilt disease at a regional scale

Pathogen invasion affected soil micro-food web structures, as shown by a survey of 100 Solanaceous plant rhizosphere soils from Jiangxi Province, China, including 50 from wilted plants infected by *R. solanacearum* (diseased soils) and 50 from asymptomatic plants (healthy soils, Fig. 1B). Pathogen invasion altered the composition of bacterial, fungal, and nematode communities (Bray–Curtis dissimilarity, *F*_1,98_ = 2.4–9.9, *P* < .05, Fig. S1A), and reduced bacterial α-diversity without affecting fungal and nematode diversity (Fig. S1B). Pathogen invasion also weakened microbial connectivity (Table S2), reducing the number of co-occurrence network links from 6535 to 3610 and particularly disrupting cross-trophic interactions between bacterivorous nematodes and *Pseudomonadota* (Figs 1C and S2). This weakening of nematode-bacteria cross-trophic interactions likely reflects pathogen-related shifts in key microbial communities. These shifts included enrichment of *Pseudomonadota* (*Alpha*-, *Beta*-, and *Gammaproteobacteria*), *Bacteroidetes*, *Gemmatimonadetes*, and the fungal phylum Mucoromycota in healthy soils, along with depletion of *Acidobacteria*, *Chloroflexi*, *Firmicutes*, and the fungal phylum Ascomycota (Figs 1D, S3, and  S4).

This regional field survey indicated that stronger nematode-bacteria cross-trophic interactions coincide with lower bacterial wilt disease. Because co-occurrence reflects association rather than causation, two explanations are plausible: (i) weakened nematode–bacteria links may permit pathogen success, or (ii) pathogen success may erode those links. To move beyond association, we assembled a 122-strain synthetic bacterial community representing dominant rhizosphere taxa of Solanaceous plants—*Pseudomonadota* (27 genera, 69 species), *Firmicutes* (6 genera, 19 species), *Actinobacteria* (5 genera, 16 species), and *Bacteroidetes* (5 genera, 18 species, Fig. 1E, Table S1)—and, in pot experiments, tested whether bacterivorous nematodes modulate pathogen populations via these interactions (Fig. 2A).

### Bacterivores reduce bacterial wilt disease by enriching rhizosphere *Pseudomonadota*

In a pot experiment, we inoculated the synthetic bacterial community alone and in combination with three nematode treatments: *Panagrolaimus* sp., *D*. *veechi*, and a mixture of both (Fig. 2A). Nematodes successfully established in all cross-trophic treatments (Fig. S5A). Nematode inoculation significantly reduced bacterial wilt incidence by 83.3% and rhizosphere *R. solanacearum* density by 11.7% (Kruskal–Wallis test: chi-square = 14.5–30.7, df = 3, *P* < .01, Fig. 2B), and reduced heterogeneity in bacterial community composition (Bray–Curtis dissimilarity, *F*_3, 62_ = 3.6, *R*^2^ = 0.15, *P* = .001, Fig. 2C). *Pseudomonadota* were enriched across all nematode treatments (ANOVA: *F*_3,61_ = 7.7, *P* < .001, Fig. 2D), with no significant changes observed in *Firmicutes*, *Bacteroidetes*, or *Actinobacteria*. *Pseudomonadota* were negatively correlated with *R. solanacearum* abundance (Pearson correlation: *n* = 72, *r* = −0.28, *P* = .02, Fig. 2E), with *Alphaproteobacteria* primarily driving the suppression effect (Pearson correlation: *n* = 72, *r* = −0.49, *P* < .001, Fig. S5B), whereas *Firmicutes* showed a positive correlation and *Bacteroidetes* were unchanged (Fig. 2E). These results point to *Pseudomonadota* having a key role in pathogen suppression within the rhizosphere.

### *Pseudomonadota* mediate cross-trophic interactions for pathogen suppression

**Figure 3:**
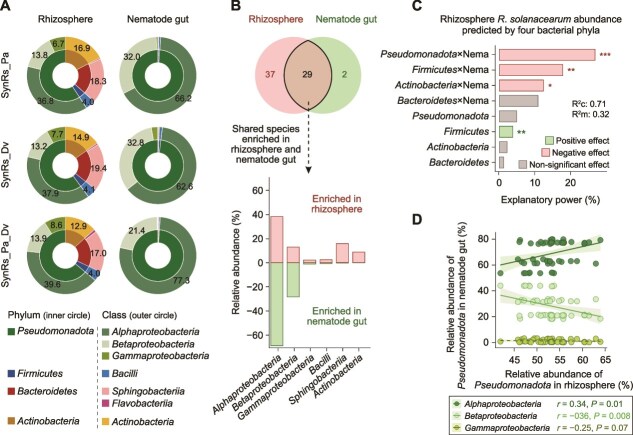
*Pseudomonadota* are the dominant taxa in nematode guts. (A) *Pseudomonadota* are more abundant in nematode guts than in the rhizosphere (*n* = 18 across three sampling times per treatment). The inner circles indicate phylum-level composition, while the outer circles indicate class-level composition. Numbers represent the relative abundance (%) of each strain. (B) Bacterial species enriched in both the rhizosphere and the nematode gut are shown; *Alphaproteobacteria* was the dominant class in both environments. Bar plots show bacterial classes enriched in the rhizosphere and the nematode gut (*n* = 18 across three sampling times per treatment). (C) Rhizosphere *Pseudomonadota* abundance is the key predictor contributing to *R. solanacearum* inhibition. This analysis was derived from a generalized linear mixed-effects model (GLMM), where the relative abundances of four phyla were treated as fixed effects, while sampling times and replicates were included as random effects (*n* = 54 across three sampling times and three treatments). Significant differences marked with “*,” “**,” and “***” indicate *P* < .05, *P* < .01, and *P* < .001, respectively. (D) *Pseudomonadota* abundance in the rhizosphere is positively correlated with *Alphaproteobacteria* abundance in nematode guts (*n* = 54 across three sampling times and three treatments). Shaded areas represent 95% confidence intervals.

The bacterial composition in the rhizosphere and nematode guts was compared, revealing that *Pseudomonadota* dominated the rhizosphere (50%) and constituted over 95% of the nematode gut microbiota (Fig. 3A). Other phyla in the rhizosphere included *Bacteroidetes* (18%), *Actinobacteria* (15%), and *Firmicutes* (4%). There were 29 shared species in both environments, 2 species exclusive to the nematode gut, and 37 species exclusive to the rhizosphere (Fig. 3B). The shared species included *Alphaproteobacteria* (5 species), *Betaproteobacteria* (10 species), *Gammaproteobacteria* (5 species), *Bacilli* (5 species), *Sphingobacteriia* (1 species), and *Actinobacteria* (3 species, Figs 3B and S5C, Table S3). *Alphaproteobacteria* were the dominant group, comprising 37%–40% of the rhizosphere community and 63%–77% of the nematode gut microbiota.

Nematode interactions with *Pseudomonadota* in the rhizosphere were associated with the greatest suppression of *R. solanacearum* (regression: *R^2^c* = 0.71, *R^2^m* = 0.32, *P* < .001, explanatory power = 26.9%, Fig. 3C). In addition, rhizosphere *Pseudomonadota* abundance correlated positively with *Alphaproteobacteria* in nematode guts (Spearman correlation: *n* = 54, *r* = 0.34, *P* = .01, Fig. 3D), indicating that the aggregate *Pseudomonadota* is largely carried by *Alphaproteobacteria*.

**Figure 4:**
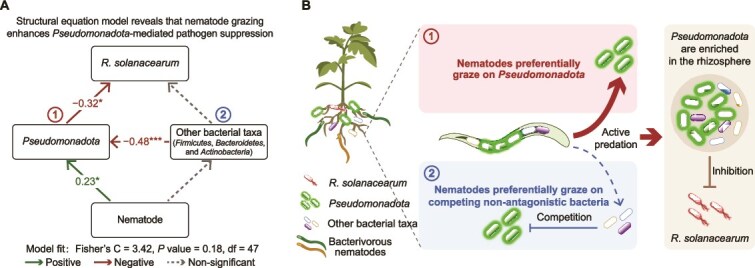
Causal pathways among nematodes, SynCom, and *R. solanacearum*. (A) A piecewise structural equation model (SEM) shows that nematode grazing enhances *Pseudomonadota*-mediated pathogen suppression. The model included nematode abundance, the relative abundances of *Pseudomonadota* and *R. solanacearum*, and the combined relative abundance of *Firmicutes*, *Bacteroidetes*, and *Actinobacteria* (*n* = 54 across three sampling times and three treatments). Sampling time and replicate were treated as random factors. Solid arrows indicate significant positive and negative paths, as indicated by the sign of the path coefficients, whereas dashed arrows indicate nonsignificant paths. The *P*-value >.05 indicates that the model fits the data very well. (B) Conceptual diagram of the two causal chains. The primary supported chain (solid) posits preferential grazing on *Pseudomonadota*, leading to their enrichment and subsequent suppression of *R. solanacearum*. The alternative chain (dashed)—which assumes no preferential grazing on nonantagonistic competitors, resulting in no enrichment and no pathogen suppression—was not supported by our data.

To confirm whether nematodes act via *Pseudomonadota*-preferential grazing or via competitor grazing, we fitted a piecewise structural equation model (SEM) that incorporated nematode abundance, *Pseudomonadota* (with *Alphaproteobacteria* as the dominant component), nonantagonistic competitors (*Firmicutes*, *Bacteroidetes*, *Actinobacteria*), and *R. solanacearum* (Fig. 4A). The SEM supported the chain in which nematodes positively affect *Pseudomonadota*, which, in turn, negatively affects *R. solanacearum*, whereas the chain via nonantagonistic competitors lacked support (Fisher’s *C* = 3.42, df = 47, *P* = .18).

### Bacterial traits predict cross-trophic interactions under pathogen stress

**Figure 5:**
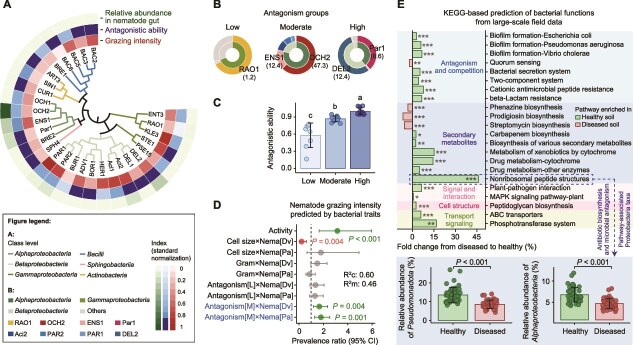
Nematodes preferentially feed on *Pseudomonadota* with moderate antagonism against plant pathogens. (A) Nematodes show stronger grazing intensity on *Pseudomonadota* compared to *Actinobacteria*, *Bacteroidetes*, and *Firmicutes* under mild *R. solanacearum* stress (*n* = 12 across two nematode species). *Pseudomonadota* have moderate antagonistic ability against *R. solanacearum* (*n* = 8 per strain). *Alphaproteobacteria* are the most abundant class in the guts of both nematode species, *Panagrolaimus* sp. and *D. veechi* (*n* = 18 across three sampling times per treatment). Heatmap values, normalized from 0 to 1, represent grazing intensity (inner circle), antagonistic ability (middle circle), and relative abundance of nematode gut microbiota (outer circle). (B) The 29 bacterial species are grouped into low, moderate, and high antagonism groups (*n* = 8 per strain). Inner circles indicate class-level composition, while outer circles show species-level composition. Numbers represent the relative abundance (%) of each strain in the nematode guts. (C) Antagonistic ability differs significantly among the three groups: Low (*n* = 8), moderate (*n* = 11), and high (*n* = 10). Different letters denote significant differences (*P* < .05). (D) Nematodes prefer bacteria with moderate pathogen antagonism. This analysis was derived from a GLMM, with bacterial traits treated as fixed effects and 29 bacterial species included as a random effect (*n* = 58 across two nematode species and 29 bacterial strains). The high antagonism group served as the reference level. Prevalence ratios > 1 or < 1 indicate positive or negative effects, respectively, and lines represent estimated marginal means ± 95% confidence intervals. (E) Predicted abundances of antagonism-related pathways are higher in healthy soils, based on PICRUSt2-inferred KEGG functional profiles (*n* = 50 per treatment). The pathway with the highest fold change—‘nonribosomal peptide structures’, mainly involved in antibiotic biosynthesis and microbial antagonism—was dominated by *Pseudomonadota*. Significant differences marked with “*,” “**,” and “***” indicate *P* < .05, *P* < .01, and *P* < .001, respectively.

*Pseudomonadota* possess several traits—such as smaller cell size, higher activity, and moderate antagonistic ability (Fig. 5D)—that likely lead to their enrichment in the nematode gut. This is based on a comprehensive analysis of physiology (activity, Gram identity, and antagonistic ability), nutrition (carbohydrate and protein content), and morphology (cell size) of 29 bacterial species shared between the rhizosphere and nematode guts (Fig. S6). We also considered the total gene number of BGCs as a predictor of secondary metabolite production. The distribution of cell size, protein content, carbohydrate content, activity, and BGC gene numbers was right-skewed, whereas antagonistic ability was left-skewed (Fig. S7). Among these species, 76% were Gram-negative (Fig. S7).

We then measured nematode grazing intensity on 29 shared bacterial species under mild *R. solanacearum* stress. Nematodes preferred *Pseudomonadota* over *Firmicutes*, *Bacteroidetes*, and *Actinobacteria* (Kruskal–Wallis test: chi-square = 44.9, df = 3, *P* < .001, Figs 5A and S8A), with *Alphaproteobacteria* being the most abundant *Pseudomonadota* class, comprising 65% of the nematode gut microbiota (Fig. 5A). *Pseudomonadota* exhibited strong antagonistic ability against *R. solanacearum* (Figs 5A and S8B). GLMM analysis revealed that bacterial traits significantly predicted nematode grazing intensity (regression: *R^2^c* = 0.71, *R^2^m* = 0.47, Fig. S8C, Table  S4). Key predictors included bacterial cell size (prevalence ratio = 27.7, *P* < .001), Gram staining characteristics (prevalence ratio = 0.1, *P* = .009), and antagonistic ability against *R. solanacearum* (prevalence ratio = 2.4, *P* = .049).

To further examine how bacterial antagonism influences nematode grazing intensity, we classified the 29 bacterial species into three groups by antagonistic ability—low, moderate, and high. The moderate group, dominated primarily by *Alphaproteobacteria* (Fig. 5B), showed significantly different antagonistic levels compared to the other groups (Kruskal–Wallis test: chi-square = 24.8, df = 2, *P* < .001, Fig. 5C). A simplified regression model further revealed that bacteria with moderate antagonism were stronger predictors of nematode grazing intensity than those with low or high antagonism (regression: *R^2^c* = 0.60, *R^2^m* = 0.46, prevalence ratio = 1.67–1.8, *P* < .01, Fig. 5D, Table S5). Consistently, the linear model relating grazing intensity to antagonistic ability revealed a unimodal pattern (Fig. S8D). These findings suggest that nematodes selectively graze on moderately antagonistic bacteria under mild *R. solanacearum* stress.

To confirm whether this trait-based selection also manifests at larger ecological scales, we analyzed KEGG-predicted functional profiles from the regional survey of bacterial communities. Healthy soils showed 45% higher abundance of antagonism-related pathways, such as nonribosomal peptide biosynthesis (ANOVA: *F*_1,98_ = 41.8, *P* < .001), compared to diseased soils, largely driven by *Alphaproteobacteria* (Fig. 5E). These soils also had a higher abundance of *Pseudomonadota* (Figs 5E and S9) and showed stronger co-occurrence between nematodes and *Pseudomonadota* (Fig. 1C). These field-scale patterns mirror those observed in controlled experiments, where nematode grazing promoted the enrichment of moderately antagonistic *Alphaproteobacteria* (Fig. 2D).

## Discussion

Cross-trophic interactions are key drivers of biodiversity and ecosystem functions [10–12]. However, the inherent complexity of microbial communities poses challenges in identifying critical microbial taxa and deciphering their interaction mechanisms. In this work, by analyzing soil microbiomes from healthy and diseased plants and the gut microbiota of nematodes, we show that *Pseudomonadota—*the most dominant and abundant bacterial phylum*—*act as keystone taxa bridging cross-trophic interactions to suppress the plant pathogen *R. solanacearum* (Fig. 4B). Specifically, under pathogen stress, nematodes preferentially graze on *Pseudomonadota*, particularly *Alphaproteobacteria* with moderate antagonistic traits, thereby enriching these bacterial taxa in the rhizosphere. This process establishes a positive feedback loop that suppresses soilborne pathogens and promotes plant health. These observations are consistent with the classic top–down control theory, in which selective predation by consumers optimizes prey community composition and enhances functional redundancy [52–54].

### *Pseudomonadota* are preferred prey for nematodes

A co-evolutionary relationship can explain nematode preference for grazing on *Pseudomonadota*. As the most diverse and abundant group in soil ecosystems [55, 56], *Pseudomonadota* have coexisted with nematodes over geological timescales [57], fostering mutual adaptations that optimize nutrient exchange. For instance, the Gram-negative cell walls of *Pseudomonadota*, rich in lipopolysaccharides, are efficiently hydrolyzed by nematode digestive enzymes, enabling rapid nutrient assimilation [58]. Concurrently, *Pseudomonadota* secrete metabolites such as vitamin B12 and polyamines, which directly enhance nematode growth and reproduction by supporting nucleic acid synthesis and cellular differentiation [59–61]. Approximately 70% of *Pseudomonadota* species support nematode fitness and promote larger body sizes, a phenomenon consistently documented across diverse nematode species [6]. In contrast, *Firmicutes* and *Actinobacteria* have limited compatibility with nematode physiology. Over 75% of species from these phyla are detrimental to nematode growth [6], largely due to their Gram-positive cell walls and frequent production of antibiotics or toxins [62–64].

Nematode preference for *Pseudomonadota* is further reinforced by sensory-driven selection mechanisms. Specifically, nematodes utilize chemosensory neurons to detect quorum-sensing molecules such as acyl-homoserine lactones, which are secreted in abundance by *Pseudomonadota* [65–67]. This molecular recognition drives preferential grazing behavior, creating a self-reinforcing ecological loop: nematode consumption enriches *Pseudomonadota* populations in the rhizosphere, which, in turn, support nematode populations through continuous nutrient provision. Unlike passive ingestion, this active selection process ensures that *Pseudomonadota* dominate the nematode diet, thereby amplifying their ecological role in pathogen suppression and nutrient cycling.

### Nematodes under pathogen stress selectively graze on *Pseudomonadota*

Nematodes exposed to soilborne pathogens selectively graze on *Pseudomonadota*. This selective feeding behavior is likely driven by two interconnected processes. The first process is plant-mediated microbial recruitment. When plants are attacked by pathogens, they release root exudates that attract beneficial microbes to the rhizosphere, a phenomenon known as the “cry for help” response [68, 69]. Among the recruited microbes, *Pseudomonadota* are particularly enriched due to their high abundance, metabolic versatility, and compatibility with plants. Some members, such as *Ochrobactrum anthropi* (OCH2), produce chitinase and β-1,3-glucanase to degrade pathogen cell walls and proteins [70, 71], whereas *Delftia lacustris* (DEL2) inhibits pathogens via volatile secondary metabolites and suppression of mycelial growth [72].

The second process is nematode-mediated microbial selection. Although nematodes actively avoid pathogens like *R. solanacearum* [37, 38], complete avoidance is difficult in the pathogen-rich rhizosphere. To reduce toxicity, nematodes selectively graze on moderately antagonistic *Pseudomonadota* (Fig. 5A), as strong antagonists can also impair nematode growth [6]. By feeding on *Pseudomonadota*, nematodes may directly benefit from their antimicrobial metabolites or indirectly by gut-mediated processes that disseminate the antagonistic bacteria into the infected rhizosphere [73–75]. A similar stress-induced feeding shift occurs in *Caenorhabditis elegans*, which selectively enriches disease-resistant *Alphaproteobacteria* under environmental stressors such as temperature fluctuations, osmotic pressure, and pathogen exposure [33, 76]. This process is regulated by the insulin signaling pathway, which modulates gut microbiota composition in response to external stress [35]. Through nematode-mediated bacterial dissemination, antagonistic *Pseudomonadota* may become more widespread in the rhizosphere, allowing them to outcompete or eliminate *R. solanacearum*, reinforcing their role in pathogen suppression.

### Implications for disease suppression, plant health, and microbial inoculant design

Recent studies have increasingly recognized that plant health is regulated not only by direct microbe–pathogen antagonism but also by complex interactions across trophic levels [13, 15, 18, 77]. Our findings contribute to this paradigm shift by demonstrating that nematode–bacteria interactions selectively enhance the persistence and functional expression of biocontrol traits. Specifically, bacteria with moderate antagonistic ability were preferentially grazed by nematodes and more effectively suppressed pathogens, suggesting that trophic feedbacks can amplify or constrain the ecological success of biocontrol agents.

These results highlight a critical limitation in current inoculant-based strategies, which typically prioritize highly antagonistic strains (e.g. *Firmicutes* and *Actinobacteria*) with limited integration into native soil food webs [63, 78]. Nonetheless, *Firmicutes*—especially spore-forming *Bacillus*—offer practical advantages as inoculants, including scalable production, long shelf life, and resilience to desiccation and temperature fluctuations, enabling broad on-farm deployment [79]. Although such strains may perform well under controlled conditions, they often fail in field applications due to poor ecological compatibility and lack of interaction with resident fauna [80, 81]. In contrast, our study identifies keystone *Pseudomonadota*, especially taxa within *Alphaproteobacteria*, that not only suppress pathogens but also engage productively with soil nematodes, as more ecologically embedded and resilient biocontrol candidates [82–84]. Prioritizing microbial agents with both functional potential and ecological connectivity, particularly those that interact with native consumers to promote persistence and dispersal, may enable more stable and scalable crop protection.

## Conclusion

Our studies identified *Pseudomonadota* as keystone-bridging taxa that connect microbial communities with higher trophic consumers, enhancing pathogen suppression and promoting plant health. This highlights the need to incorporate cross-trophic interactions into biocontrol strategies for more resilient pathogen control and to move beyond conventional approaches that rely on certain single-strain microbial inoculants, which often suffer from poor establishment and inconsistent performance. Additionally, the functional traits of biocontrol agents, such as antagonistic activity and metabolic activity, shape their interactions with nematode predators. By selectively targeting microbes based on these traits, nematodes act as central nodes in the soil micro-food web, filtering microbial community and antagonistic functions from the microscale to the regional scale. These findings call for developing biocontrol agents from taxa that combine antagonistic efficacy and trophic compatibility to achieve stable, long-term pathogen suppression.

## Supplementary Material

Supplementary_information_2_5_wrag011

## Data Availability

The 16S and 18S rRNA gene amplicon sequencing data from the 100 regional-scale sampling sites, the 16S rRNA gene sequences of the 122 bacterial strains used in the synthetic community pot experiment, and all data and code used in this study are available via Figshare at https://figshare.com/s/dd4a0c448c393b7b4efd (ref. 85).
