# Supplementary material for: Pseudomonadota bridge cross-trophic interactions to suppress plant pathogens
Source: ISME J. 2026 Jan 28;20(1):wrag011. doi: 10.1093/ismejo/wrag011 (PMC12904284; doi:10.1093/ismejo/wrag011)
Supplement: Supplementary_information_2_5_wrag011 [file supplementary_information_2_5_wrag011.pdf]

Supplementary Information for

***Pseudomonadota* bridge cross-trophic interactions to suppress plant pathogens**

Huiyu Chuai[1,2,3#], Gen Li[1,2,3#], Luchen Tao[1,2], Lei Ouyang[1,2], Ruihan Ruan[1,2], Zhong Wei[3], Joann Whalen[4,5], Uffe N. Nielsen[6], Ting Liu[1,2*], Huixin Li[1,2]

[1]Asia Hub on Agriculture, Sanya Institute of Nanjing Agricultural University, Sanya, Hainan 572024, China
[2]Soil Ecology Lab, College of Resources and Environmental Sciences, Nanjing Agricultural University, Nanjing, Jiangsu 210095, China
[3]Jiangsu Collaborative Innovation Center for Solid Organic Waste Resource Utilization, Nanjing Agricultural University, Nanjing, Jiangsu 210095, China
[4]Department of Natural Resource Sciences, McGill University, Montreal, QC H9X 3V9, Canada
[5]Chair of Soil Science, Mohammed VI Polytechnic University, Ben Guerir, Marrakech–Safi 43150, Morocco
[6]Hawkesbury Institute for the Environment, Western Sydney University, Penrith, NSW 2751, Australia

[*] Corresponding author. College of Resources and Environmental Sciences, Nanjing Agricultural University, No. 1 Weigang, Nanjing, Jiangsu Province 210095, China. E-mail: ting.liu@njau.edu.cn
[#] Huiyu Chuai and Gen Li contributed equally to this work.

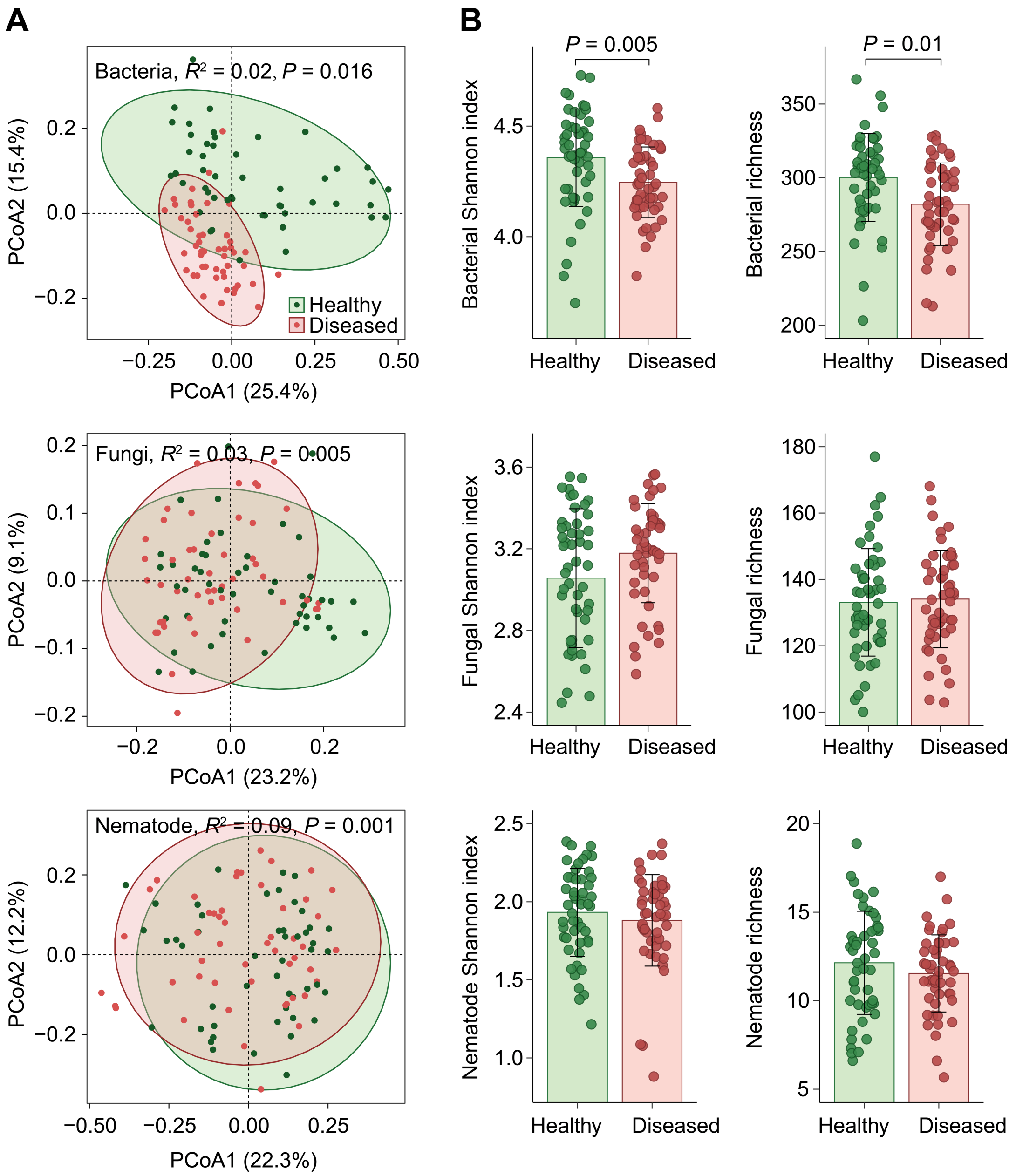

Supplementary Figure 1. Microbial community composition and diversity in soils from healthy and *Ralstonia solanacearum*-infected plants across a regional-scale survey. (A) Principal coordinate analysis (PCoA) of bacterial, fungal, and nematode communities based on Bray-Curtis dissimilarities (n = 50 per treatment). (B) α-diversity of bacterial, fungal, and nematode communities in healthy and diseased soils (n = 50 per treatment).

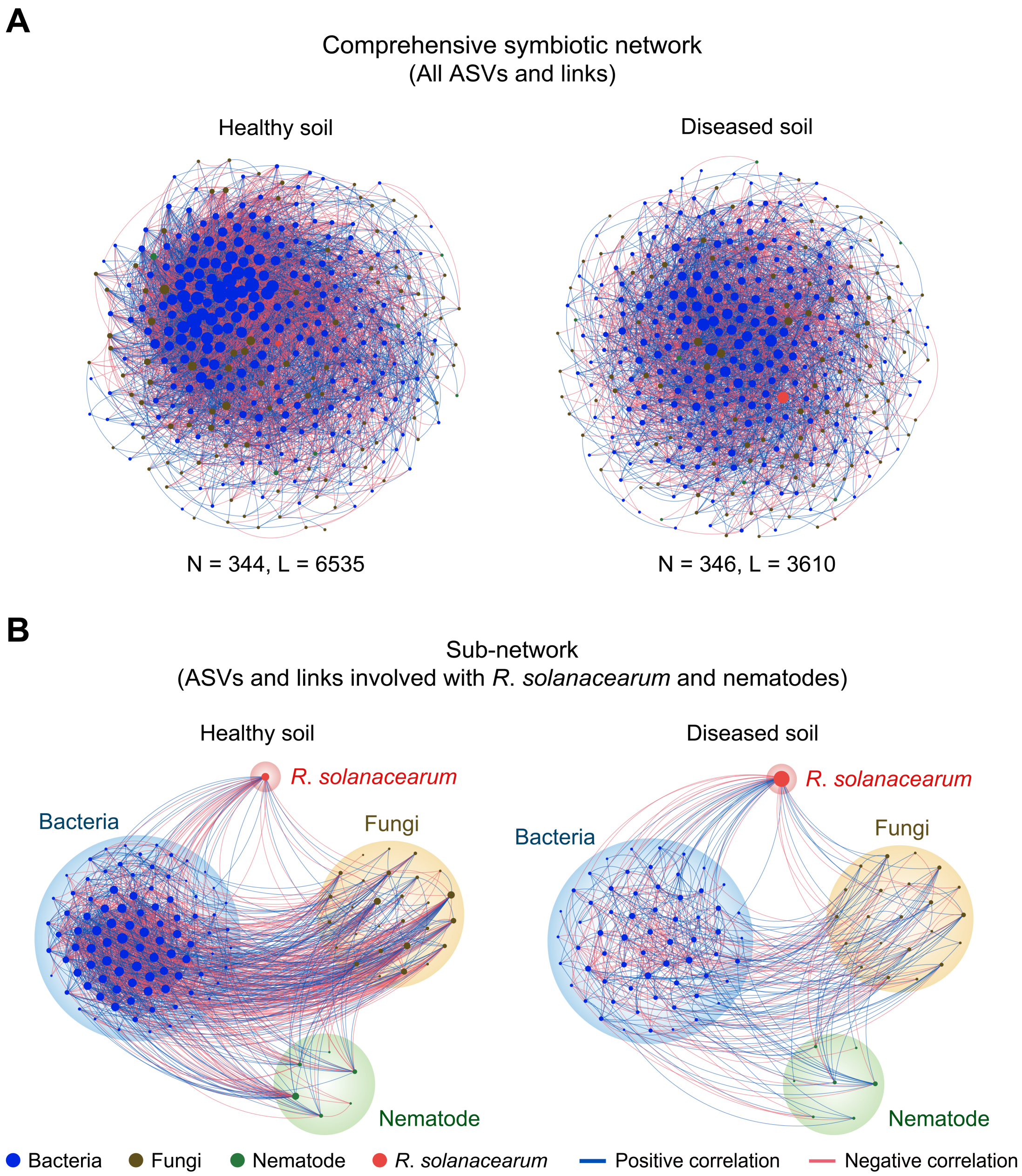

Supplementary Figure 2. Pathogen infection simplifies the co-occurrence structure of soil micro-food webs. (A) Co-occurrence networks based on SparCC ($|r| > 0.3$, $P < 0.05$) show reduced network complexity under pathogen infection (n = 50 per treatment). Only connected nodes are shown, representing genus-level taxa (ASVs assigned at genus level), with node size proportional to the number of associations. "N" and "L" denote the number of nodes and links, respectively. (B) Sub-networks focused on interactions involving *R. solanacearum* and nematodes (n = 50 per treatment), constructed using the same criteria as in (A).

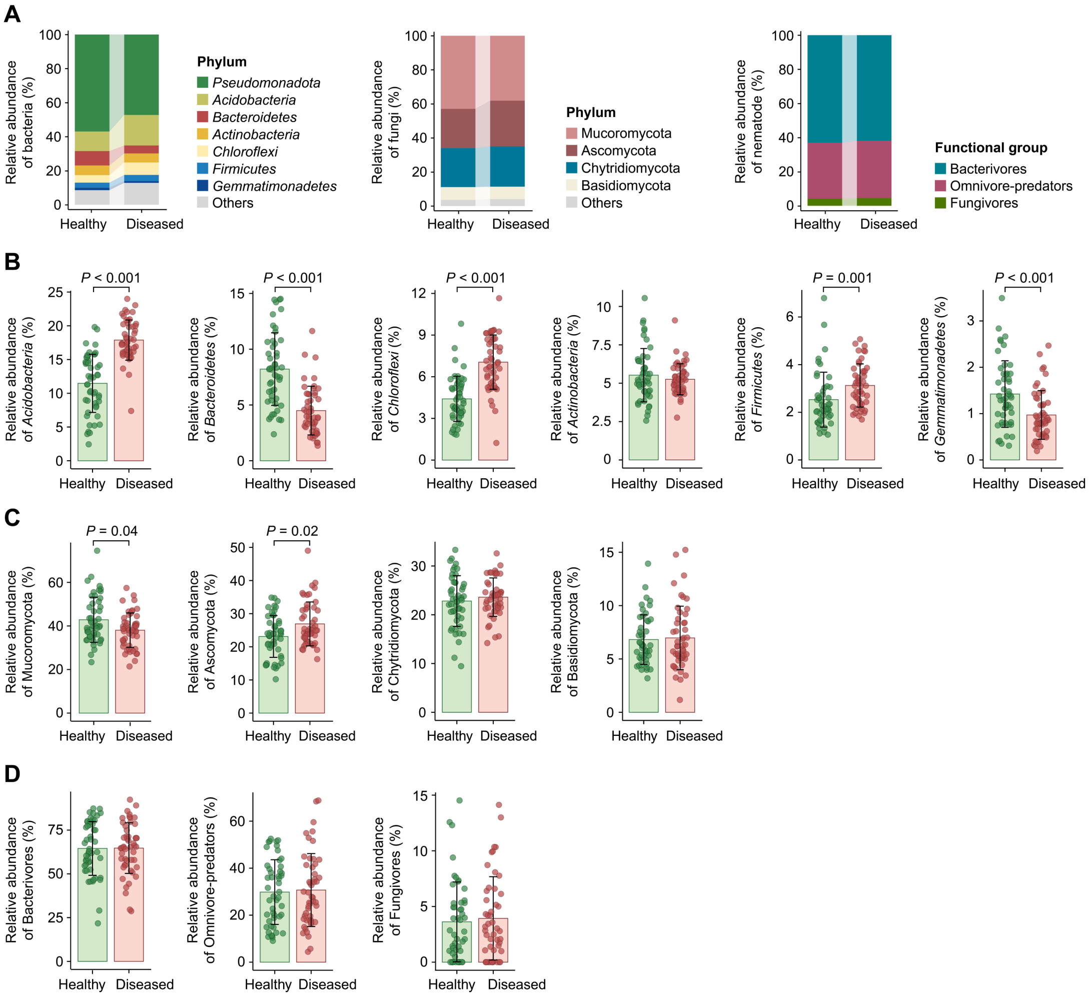

Supplementary Figure 3. Taxonomic shifts in soil bacterial, fungal, and nematode communities under pathogen infection. (A) Taxonomic composition of soil bacteria, fungi, and nematodes in healthy and pathogen-infected soils (n = 50 per treatment). (B) Relative abundances of major bacterial phyla (n = 50 per treatment). (C) Relative abundances of dominant fungal phyla (n = 50 per treatment). (D) Relative abundances of key nematode functional groups (n = 50 per treatment).

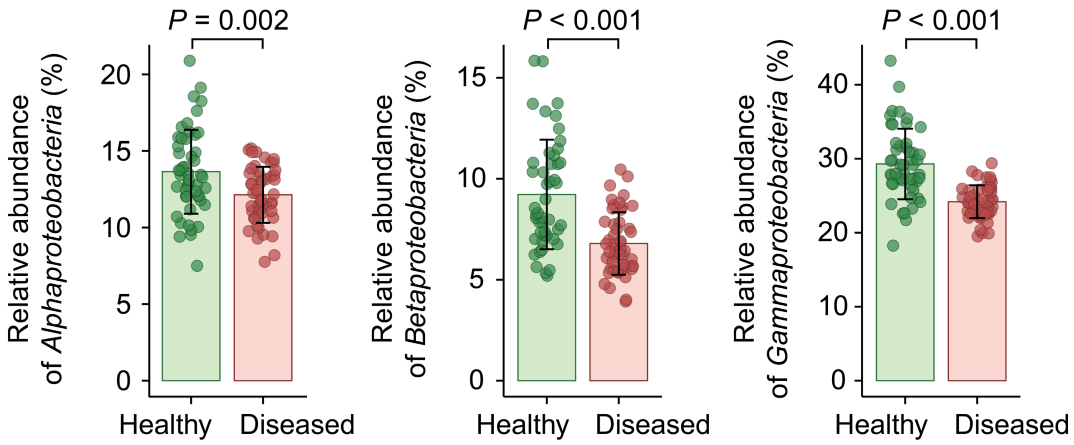

Supplementary Figure 4. Pathogen infection reduces the relative abundances of *Alphaproteobacteria*, *Betaproteobacteria*, and *Gammaproteobacteria* (n = 50 per treatment).

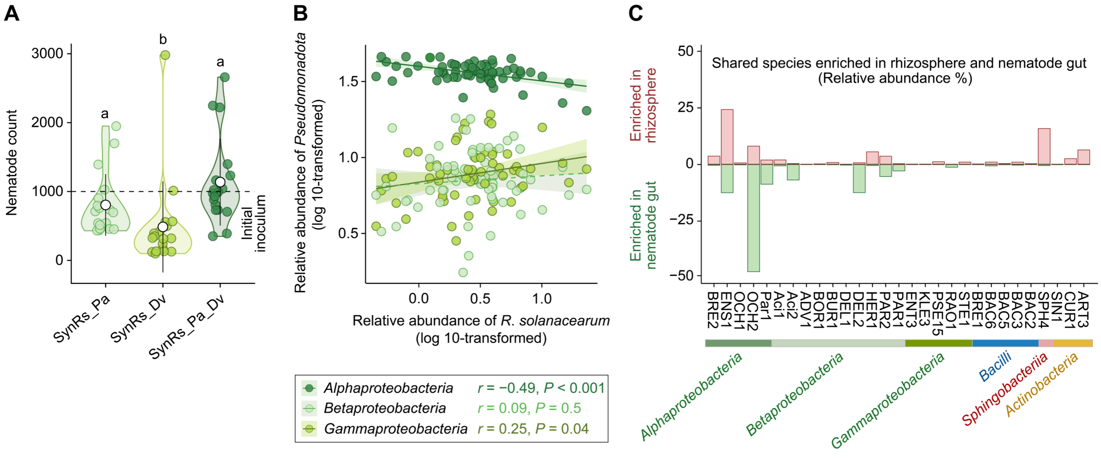

Supplementary Figure 5. *Alphaproteobacteria* as predictors of cross-trophic microbial interactions related to *R. solanacearum* suppression. (A) Nematode densities across the treatments (n = 18 across three sampling times per treatment). The dashed line indicates the initial inoculum. Different letters indicate significant differences among treatments ($P < 0.05$). (B) Negative correlation between rhizosphere *Alphaproteobacteria* and *R. solanacearum* abundance (n = 72 across three sampling times and four treatments). Shaded areas represent 95% confidence intervals. (C) Bacterial species enriched in the rhizosphere and nematode guts (n = 18 across three sampling times per treatment).

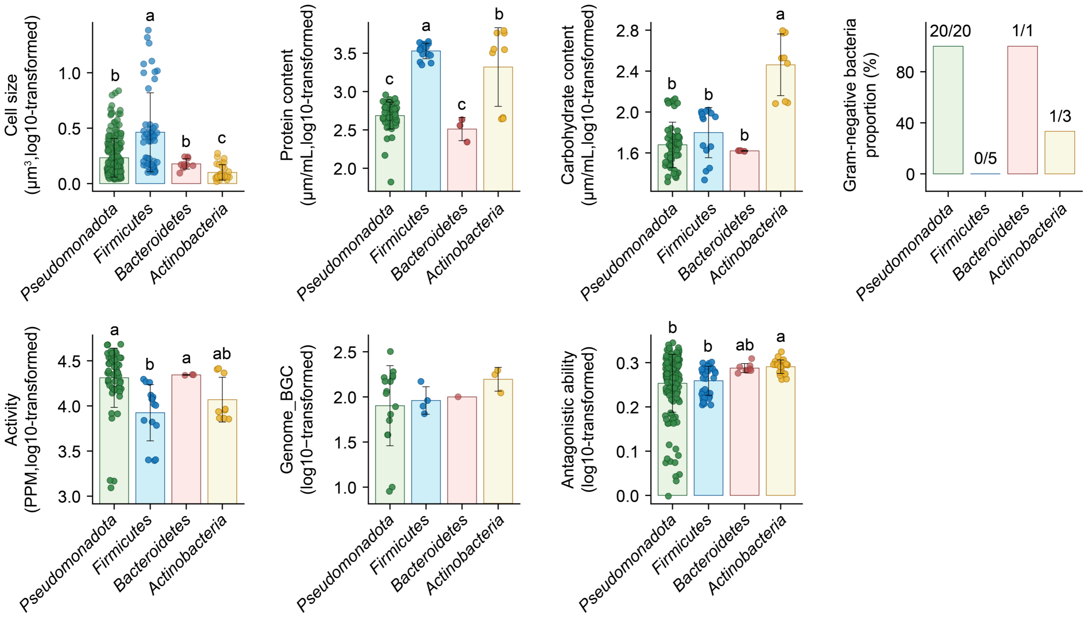

Supplementary Figure 6. Comparison of bacterial traits across four phyla.

Different letters indicate significant differences among groups ($P < 0.05$).

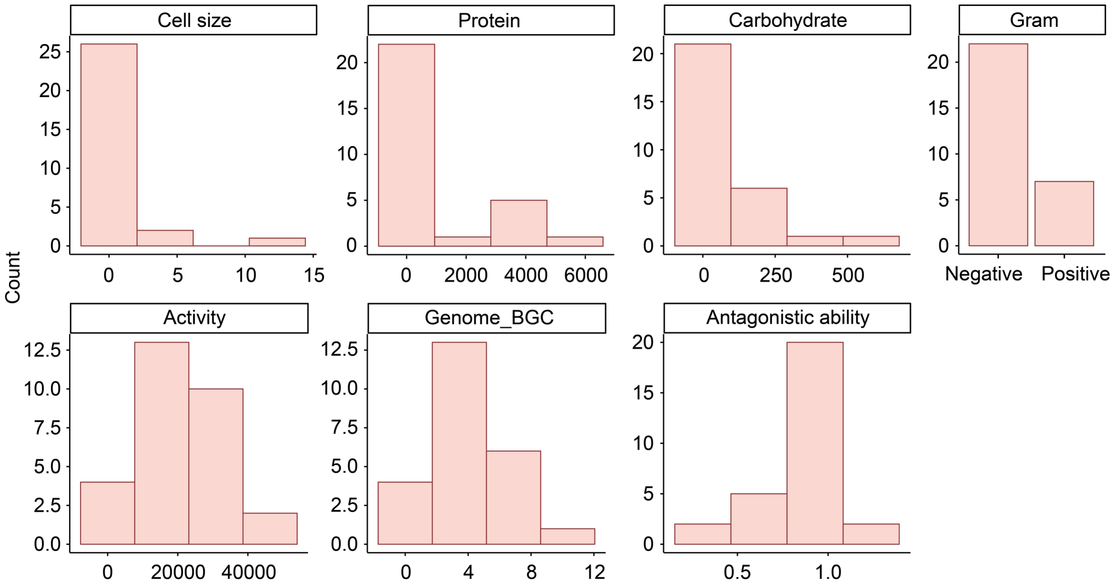

Supplementary Figure 7. Distribution of bacterial traits among the 29 bacterial species.

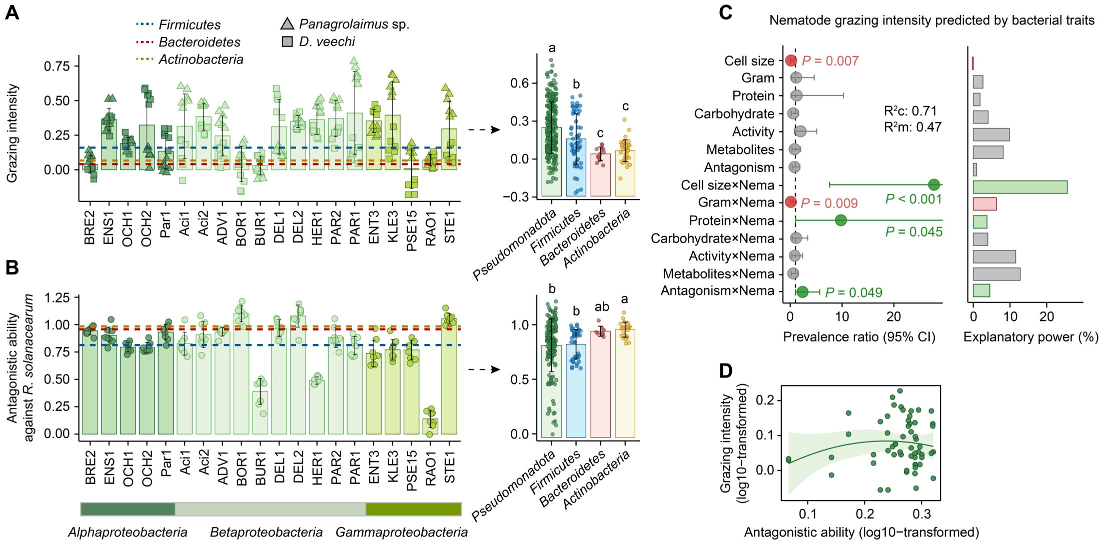

Supplementary Figure 8. Nematodes preferentially feed on antagonistic *Pseudomonadota* under mild pathogen stress. (A) Nematodes graze more on *Pseudomonadota* than on *Firmicutes*, *Bacteroidetes*, or *Actinobacteria* under mild *R. solanacearum* stress (n = 12 across two nematode species). Grazing intensity is shown for 29 shared bacterial strains (20 *Pseudomonadota* strains; 9 other strains averaged, indicated by dashed lines). Right panel: phylum-level comparison. Different letters indicate significant differences among groups ($P < 0.05$). (B) *Pseudomonadota* show strong antagonistic ability against *R. solanacearum* (n = 8 per strain). Bars are colored by different *Pseudomonadota* classes. Right panel: phylum-level comparison. (C) Bacterial cell size , Gram staining characteristics, and antagonistic ability were key predictors of nematode grazing intensity, based on a GLMM (n = 58 across two nematode species and 29 bacterial strains). In the model, bacterial traits were treated as fixed effects, and the 29 bacterial species were included as a random effect. Right panel: predictor contributions. Prevalence ratios > 1 or < 1 indicate positive or negative effects, respectively, and lines represent estimated marginal means ± 95% confidence intervals. (D) Nonlinear relationship between grazing intensity and antagonistic ability, with a peak at moderate levels (n = 58 across two nematode species and 29 bacterial strains). Shaded area indicates 95% confidence intervals.

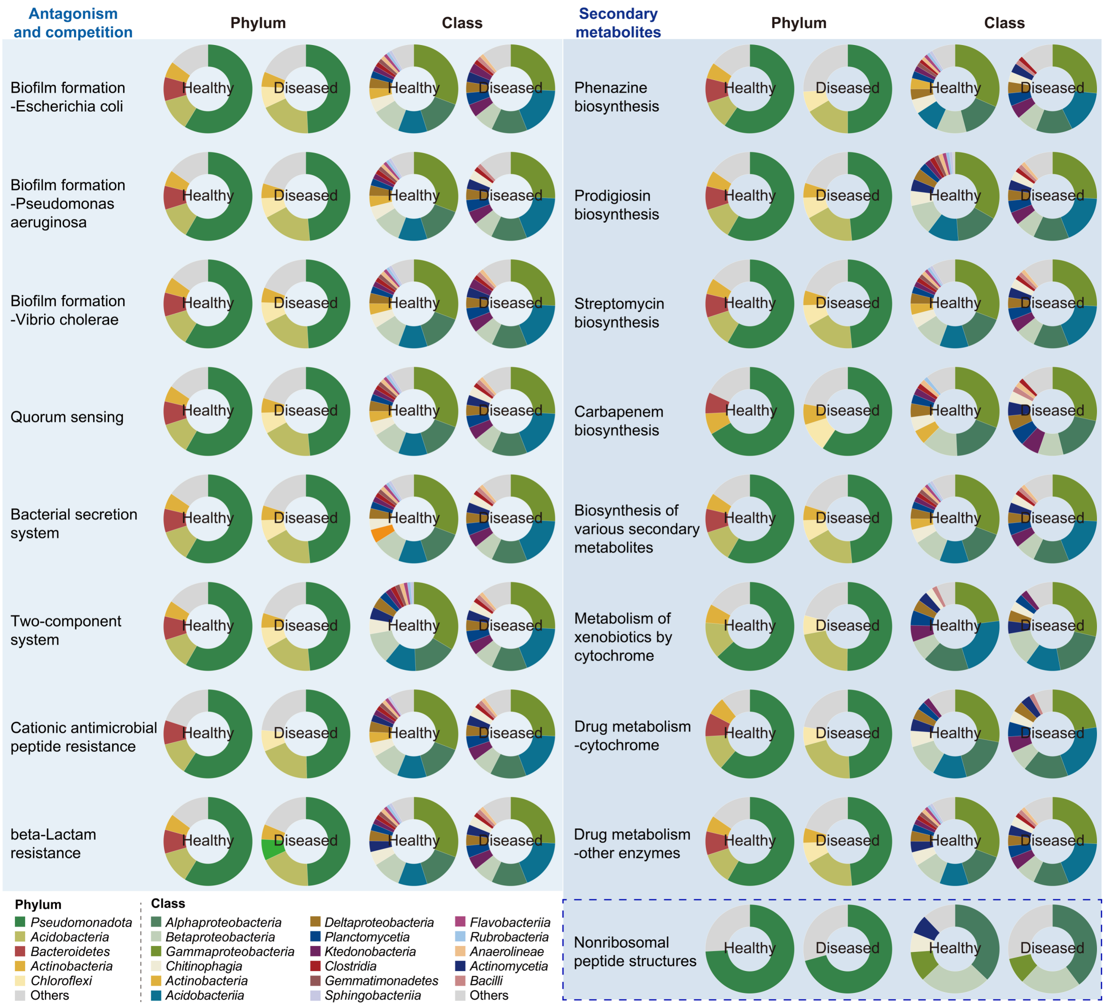

Supplementary Figure 9. Predicted abundances of antagonism-related pathways were higher in healthy soils. The analysis is based on PICRUSt2-inferred KEGG functional profiles (n = 50 per treatment).

Supplementary Table 1. Taxonomic information of the 122-member synthetic bacterial community.

Spe. ID	Phylum	Class	Genus	Species
ACH1	*Pseudomonadota*	*Betaproteobacteria*	*Achromobacter*	*Achromobacter marplatensis*
ACH2	*Pseudomonadota*	*Betaproteobacteria*	*Achromobacter*	*Achromobacter mucicolens*
Aci1	*Pseudomonadota*	*Betaproteobacteria*	*Acidovorax*	*Acidovorax avenae*
Aci2	*Pseudomonadota*	*Betaproteobacteria*	*Acidovorax*	*Acidovorax wautersii*
ACI1	*Pseudomonadota*	*Gammaproteobacteria*	*Acinetobacter*	*Acinetobacter baumannii*
ACI2	*Pseudomonadota*	*Gammaproteobacteria*	*Acinetobacter*	*Acinetobacter beijerinckii*
ACI3	*Pseudomonadota*	*Gammaproteobacteria*	*Acinetobacter*	*Acinetobacter bereziniae*
ACI4	*Pseudomonadota*	*Gammaproteobacteria*	*Acinetobacter*	*Acinetobacter calcoaceticus*
ACI5	*Pseudomonadota*	*Gammaproteobacteria*	*Acinetobacter*	*Acinetobacter gyllenbergii*
ACI6	*Pseudomonadota*	*Gammaproteobacteria*	*Acinetobacter*	*Acinetobacter johnsonii*
ACI7	*Pseudomonadota*	*Gammaproteobacteria*	*Acinetobacter*	*Acinetobacter kookii*
ACI8	*Pseudomonadota*	*Gammaproteobacteria*	*Acinetobacter*	*Acinetobacter oleivorans*
ACI9	*Pseudomonadota*	*Gammaproteobacteria*	*Acinetobacter*	*Acinetobacter pittii*
ACI10	*Pseudomonadota*	*Gammaproteobacteria*	*Acinetobacter*	*Acinetobacter soli*
ACI11	*Pseudomonadota*	*Gammaproteobacteria*	*Acinetobacter*	*Acinetobacter vivianii*
ADV1	*Pseudomonadota*	*Betaproteobacteria*	*Advenella*	*Advenella kashmirensis*
BOR1	*Pseudomonadota*	*Betaproteobacteria*	*Bordetella*	*Bordetella petrii*
BRE2	*Pseudomonadota*	*Alphaproteobacteria*	*Brevundimonas*	*Brevundimonas diminuta*
BUR1	*Pseudomonadota*	*Betaproteobacteria*	*Burkholderia*	*Burkholderia cenocepacia*
CUP1	*Pseudomonadota*	*Betaproteobacteria*	*Cupriavidus*	*Cupriavidus alkaliphilus*
CUP2	*Pseudomonadota*	*Betaproteobacteria*	*Cupriavidus*	*Cupriavidus necator*
CUP3	*Pseudomonadota*	*Betaproteobacteria*	*Cupriavidus*	*Cupriavidus yeoncheonensis*
DEL1	*Pseudomonadota*	*Betaproteobacteria*	*Delftia*	*Delftia acidovorans*
DEL2	*Pseudomonadota*	*Betaproteobacteria*	*Delftia*	*Delftia lacustris*
DYE1	*Pseudomonadota*	*Gammaproteobacteria*	*Dyella*	*Dyella marensis*
ENS1	*Pseudomonadota*	*Alphaproteobacteria*	*Ensifer*	*Ensifer adhaerens*
ENT1	*Pseudomonadota*	*Gammaproteobacteria*	*Enterobacter*	*Enterobacter cancerogenus*
ENT2	*Pseudomonadota*	*Gammaproteobacteria*	*Enterobacter*	*Enterobacter ludwigii*
ENT3	*Pseudomonadota*	*Gammaproteobacteria*	*Enterobacter*	*Enterobacter mori*
ESC1	*Pseudomonadota*	*Gammaproteobacteria*	*Escherichia*	*Atlantibacter hermannii*
FIC1	*Pseudomonadota*	*Gammaproteobacteria*	*Kluyvera*	*Kluyvera georgiana*
HER1	*Pseudomonadota*	*Betaproteobacteria*	*Herbaspirillum*	*Herbaspirillum rubrisubalbicans*
KLE1	*Pseudomonadota*	*Gammaproteobacteria*	*Klebsiella*	*Klebsiella michiganensis*
KLE2	*Pseudomonadota*	*Gammaproteobacteria*	*Klebsiella*	*Klebsiella pneumoniae*
KLE3	*Pseudomonadota*	*Gammaproteobacteria*	*Klebsiella*	*Klebsiella variicola*
KOS1	*Pseudomonadota*	*Gammaproteobacteria*	*Kosakonia*	*Kosakonia cowanii*
KOS2	*Pseudomonadota*	*Gammaproteobacteria*	*Kosakonia*	*Kosakonia sacchari*
OCH1	*Pseudomonadota*	*Alphaproteobacteria*	*Ochrobactrum*	*Brucella pseudogrignonensis*
OCH2	*Pseudomonadota*	*Alphaproteobacteria*	*Ochrobactrum*	*Ochrobactrum anthropi*
PAR1	*Pseudomonadota*	*Betaproteobacteria*	*Paraburkholderia*	*Paraburkholderia oxyphila*
PAR2	*Pseudomonadota*	*Betaproteobacteria*	*Paraburkholderia*	*Paraburkholderia phenoliruptrix*
Par1	*Pseudomonadota*	*Alphaproteobacteria*	*Paracoccus*	*Paracoccus yeei*
PRO1	*Pseudomonadota*	*Gammaproteobacteria*	*Providencia*	*Providencia rustigianii*
PRO2	*Pseudomonadota*	*Gammaproteobacteria*	*Providencia*	*Providencia vermicola*
PSE1	*Pseudomonadota*	*Gammaproteobacteria*	*Pseudomonas*	*Pseudomonas baetica*
PSE2	*Pseudomonadota*	*Gammaproteobacteria*	*Pseudomonas*	*Pseudomonas brassicacearum*
PSE3	*Pseudomonadota*	*Gammaproteobacteria*	*Pseudomonas*	*Pseudomonas chlororaphis*
PSE4	*Pseudomonadota*	*Gammaproteobacteria*	*Pseudomonas*	*Pseudomonas donghuensis*
PSE5	*Pseudomonadota*	*Gammaproteobacteria*	*Pseudomonas*	*Pseudomonas fluorescens*
PSE6	*Pseudomonadota*	*Gammaproteobacteria*	*Pseudomonas*	*Pseudomonas koreensis*
PSE7	*Pseudomonadota*	*Gammaproteobacteria*	*Pseudomonas*	*Pseudomonas mohnii*
PSE8	*Pseudomonadota*	*Gammaproteobacteria*	*Pseudomonas*	*Pseudomonas monteilii*
PSE9	*Pseudomonadota*	*Gammaproteobacteria*	*Pseudomonas*	*Pseudomonas moorei*
PSE10	*Pseudomonadota*	*Gammaproteobacteria*	*Pseudomonas*	*Pseudomonas protegens*
PSE11	*Pseudomonadota*	*Gammaproteobacteria*	*Pseudomonas*	*Pseudomonas putida*
PSE12	*Pseudomonadota*	*Gammaproteobacteria*	*Pseudomonas*	*Pseudomonas thivervalensis*
PSE13	*Pseudomonadota*	*Gammaproteobacteria*	*Pseudomonas*	*Pseudomonas vancouverensis*
PSE14	*Pseudomonadota*	*Gammaproteobacteria*	*Pseudomonas*	*Pseudomonas vranovensis*
PSE15	*Pseudomonadota*	*Gammaproteobacteria*	*Pseudoxanthomonas*	*Pseudoxanthomonas mexicana*
RAL1	*Pseudomonadota*	*Betaproteobacteria*	*Ralstonia*	*Ralstonia mannitolilytica*

RAL5	*Pseudomonadota*	*Betaproteobacteria*	*Ralstonia*	*Ralstonia mannitolilytica*
RAL2	*Pseudomonadota*	*Betaproteobacteria*	*Ralstonia*	*Ralstonia pickettii*
RAL3	*Pseudomonadota*	*Betaproteobacteria*	*Ralstonia*	*Ralstonia pickettii*
RAL4	*Pseudomonadota*	*Betaproteobacteria*	*Ralstonia*	*Ralstonia taiwanensis*
RAO1	*Pseudomonadota*	*Gammaproteobacteria*	*Raoultella*	*Raoultella ornithinolytica*
STE1	*Pseudomonadota*	*Gammaproteobacteria*	*Stenotrophomonas*	*Stenotrophomonas acidaminiphila*
STE2	*Pseudomonadota*	*Gammaproteobacteria*	*Stenotrophomonas*	*Stenotrophomonas bentonitica*
STE3	*Pseudomonadota*	*Gammaproteobacteria*	*Stenotrophomonas*	*Stenotrophomonas maltophilia*
VAR1	*Pseudomonadota*	*Betaproteobacteria*	*Variovorax*	*Variovorax guangxiensis*
BAC1	*Firmicutes*	*Bacilli*	*Bacillus*	*Bacillus amyloliquefaciens*
BAC2	*Firmicutes*	*Bacilli*	*Bacillus*	*Bacillus aryabhattai*
BAC3	*Firmicutes*	*Bacilli*	*Bacillus*	*Bacillus koreensis*
BAC4	*Firmicutes*	*Bacilli*	*Bacillus*	*Bacillus mycoides*
BAC5	*Firmicutes*	*Bacilli*	*Bacillus*	*Bacillus nealsonii*
BAC6	*Firmicutes*	*Bacilli*	*Bacillus*	*Bacillus shackletonii*
BAC7	*Firmicutes*	*Bacilli*	*Bacillus*	*Bacillus subtilis*
BAC8	*Firmicutes*	*Bacilli*	*Bacillus*	*Bacillus velezensis*
BRE1	*Firmicutes*	*Bacilli*	*Brevibacillus*	*Brevibacillus parabrevis*
FAL1	*Firmicutes*	*Bacilli*	*Falsibacillus*	*Falsibacillus pallidus*
LYS1	*Firmicutes*	*Bacilli*	*Lysinibacillus*	*Lysinibacillus pakistanensis*
LYS2	*Firmicutes*	*Bacilli*	*Lysinibacillus*	*Lysinibacillus sphaericus*
PAE1	*Firmicutes*	*Bacilli*	*Paenibacillus*	*Paenibacillus cineris*
PAE2	*Firmicutes*	*Bacilli*	*Paenibacillus*	*Paenibacillus illinoisensis*
PAE3	*Firmicutes*	*Bacilli*	*Paenibacillus*	*Paenibacillus rhizosphaerae*
PAE4	*Firmicutes*	*Bacilli*	*Paenibacillus*	*Paenibacillus taichungensis*
PAE5	*Firmicutes*	*Bacilli*	*Paenibacillus*	*Paenibacillus tundrae*
PAE6	*Firmicutes*	*Bacilli*	*Paenibacillus*	*Paenibacillus xylanexedens*
STA1	*Firmicutes*	*Bacilli*	*Staphylococcus*	*Staphylococcus hominis*
CHR1	*Bacteroidetes*	*Flavobacteriia*	*Chryseobacterium*	*Chryseobacterium arthrosphaerae*
CHR2	*Bacteroidetes*	*Flavobacteriia*	*Chryseobacterium*	*Chryseobacterium cucumeris*
CHR3	*Bacteroidetes*	*Flavobacteriia*	*Chryseobacterium*	*Chryseobacterium daecheongense*
CHR4	*Bacteroidetes*	*Flavobacteriia*	*Chryseobacterium*	*Chryseobacterium geocarposphaerae*
CHR5	*Bacteroidetes*	*Flavobacteriia*	*Chryseobacterium*	*Chryseobacterium sediminis*
CHR6	*Bacteroidetes*	*Flavobacteriia*	*Chryseobacterium*	*Chryseobacteriumrhizoplanae*
EMP1	*Bacteroidetes*	*Flavobacteriia*	*Empedobacter*	*Empedobacter brevis*
EMP2	*Bacteroidetes*	*Flavobacteriia*	*Empedobacter*	*Empedobacter falsenii*
FLA1	*Bacteroidetes*	*Flavobacteriia*	*Flavobacterium*	*Flavobacterium anhuiense*
FLA2	*Bacteroidetes*	*Flavobacteriia*	*Flavobacterium*	*Flavobacterium denitrificans*
FLA3	*Bacteroidetes*	*Flavobacteriia*	*Flavobacterium*	*Flavobacterium johnsoniae*
FLA4	*Bacteroidetes*	*Flavobacteriia*	*Flavobacterium*	*Flavobacterium nitrogenifigens*
FLA5	*Bacteroidetes*	*Flavobacteriia*	*Flavobacterium*	*Flavobacterium naphthae*
FLA6	*Bacteroidetes*	*Flavobacteriia*	*Flavobacterium*	*Myroides anatoliensis*
SPH1	*Bacteroidetes*	*Sphingobacteriia*	*Sphingobacterium*	*Sphingobacterium ginsenosidimutans*
SPH2	*Bacteroidetes*	*Sphingobacteriia*	*Sphingobacterium*	*Sphingobacterium mizutaii*
SPH3	*Bacteroidetes*	*Sphingobacteriia*	*Sphingobacterium*	*Sphingobacterium thalpophilum*
SPH4	*Bacteroidetes*	*Sphingobacteriia*	*Sphingomonas*	*Sphingomonas yabuuchiae*
ART1	*Actinobacteria*	*Actinobacteria*	*Arthrobacter*	*Arthrobacter bambusae*
ART2	*Actinobacteria*	*Actinobacteria*	*Arthrobacter*	*Arthrobacter cupressi*
ART3	*Actinobacteria*	*Actinobacteria*	*Arthrobacter*	*Arthrobacter globiformis*
ART4	*Actinobacteria*	*Actinobacteria*	*Arthrobacter*	*Pseudarthrobacter defluvii*
ART5	*Actinobacteria*	*Actinobacteria*	*Arthrobacter*	*Pseudarthrobacter niigatensis*
ART6	*Actinobacteria*	*Actinobacteria*	*Arthrobacter*	*Glutamicibacter nicotianae*
ART7	*Actinobacteria*	*Actinobacteria*	*Arthrobacter*	*Pseudarthrobacter equi*
ART8	*Actinobacteria*	*Actinobacteria*	*Arthrobacter*	*Paenarthrobacter nicotinovorans*
ART9	*Actinobacteria*	*Actinobacteria*	*Arthrobacter*	*Paenarthrobacter ureafaciens*
CUR1	*Actinobacteria*	*Actinobacteria*	*Curtobacterium*	*Curtobacterium citreum*
MIC1	*Actinobacteria*	*Actinobacteria*	*Microbacterium*	*Microbacterium hydrocarbonoxydans*
MIC2	*Actinobacteria*	*Actinobacteria*	*Microbacterium*	*Microbacterium laevaniformans*
MIC3	*Actinobacteria*	*Actinobacteria*	*Microbacterium*	*Microbacterium testaceum*
MIC4	*Actinobacteria*	*Actinobacteria*	*Microbacterium*	*Microbacterium thalassium*
RHO1	*Actinobacteria*	*Actinobacteria*	*Rhodococcus*	*Rhodococcus agglutinans*
SIN1	*Actinobacteria*	*Actinobacteria*	*Sinomonas*	*Sinomonas flava*

Supplementary Table 2. Topological properties of comprehensive symbiotic networks and their sub-networks.

Classification	Group	Healthy soil	Diseased soil
Comprehensive symbiotic network	Node number	344	346
	Edge number	6535	3610
	Average degree	38.00	20.87
	Average path length	2.21	2.45
	Positive correlation (%)	51.74	54.29
	Negative correlation (%)	48.26	45.71
Sub-network	Node number	129	93
	Edge number	1824	504
	Average degree	28.28	10.84
	Average path length	2.02	2.36
	Positive correlation (%)	50.71	57.14
	Negative correlation (%)	49.29	42.86

Supplementary Table 3. Counts and relative abundances of bacterial species in the rhizosphere and nematode guts. Bacterial species enriched in the rhizosphere and nematode guts are highlighted in red and green, respectively.

Rhizosphere specific species (n = 37)	Relative abundance (%)	Nematode guts specific species (n = 2)	Relative abundance (%)	Rhizosphere and nematode guts shared species (n = 29)	Relative abundance (%)
ART1	0.73	ACH1	0.39	ART3	6.26, 0.02
ART2	2.25	ACI5	0.01	CUR1	2.42, 0.01
ART5	0.37			SIN1	0.01, 0.03
ART6	0.06			SPH4	15.73, 0.43
ART7	0.70			BAC2	0.22, 0.01
ART9	1.37			BAC3	0.89, 0.23
MIC1	0.02			BAC5	0.29, 0.05
MIC3	0.69			BAC6	0.91, 0.53
CHR4	0.47			BRE1	0.10, 0.02
FLA1	0.06			BRE2	3.56, 0.28
FLA2	0.02			ENS1	24.13, 12.38
FLA3	0.17			OCH1	0.55, 0.11
SPH1	0.14			OCH2	7.97, 47.24
SPH2	0.94			Par1	1.84, 8.64
SPH3	1.40			Aci1	1.87, 0.53
BAC1	0.02			Aci2	0.29, 6.77
BAC4	0.01			ADV1	0.01, 0.01
FAL1	0.74			BOR1	0.11, 0.01
LYS1	0.14			BUR1	0.76, 0.05
PAE1	0.20			DEL1	0.01, 0.20
PAE2	0.07			DEL2	0.59, 12.37
PAE5	0.40			HER1	5.44, 0.41
STA1	0.02			PAR2	3.51, 5.23
ACH2	0.37			PAR1	0.22, 2.77
CUP1	0.06			ENT3	0.05, 0.01
CUP2	0.06			KLE3	0.05, 0.01
VAR1	0.30			PSE15	1.07, 0.02
ACI9	0.01			RAO1	0.01, 1.15
ENT1	0.02			STE1	0.91, 0.01
KLE2	0.07				
PSE11	0.22				
PSE2	0.02				
PSE3	0.01				
PSE7	0.02				
STE2	4.99				
STE3	0.19				
R. solanacearum	2.84				

Supplementary Table 4. Nematode grazing intensity predicted by seven bacterial traits using a GLMM (n = 58 across two nematode species and 29 bacterial strains). Seven bacterial traits were treated as fixed effects, with bacterial species treated as a random effect.

**Parameter**	**Prevalence ratio**	**Standard error**	**Explanatory power**	***P* value**
**Model fit: Conditional R^2 = 0.71, Marginal R^2 = 0.47**				
Cell size	0.18	0.11	−0.07	0.007
Gram	1.14	0.78	2.69	0.853
Protein	1.27	1.30	1.89	0.816
Carbohydrate	0.58	0.29	4.08	0.293
Activity	2.01	0.92	9.88	0.137
Metabolites	0.84	0.36	8.16	0.688
Antagonism	0.83	0.33	0.93	0.642
Cell size × Nematode	27.73	17.59	25.61	< 0.001
Gram × Nematode	0.13	0.09	6.30	0.009
Protein × Nematode	9.78	10.68	3.75	0.045
Carbohydrate × Nematode	1.13	0.61	3.98	0.824
Activity × Nematode	0.88	0.43	11.58	0.789
Metabolites × Nematode	0.59	0.27	12.80	0.263
Antagonism × Nematode	2.39	1.01	4.51	0.049

Supplementary Table 5. Nematode grazing intensity predicted by four bacterial traits using a GLMM (n = 58 across two nematode species and 29 bacterial strains). Four bacterial traits were treated as fixed effects, with bacterial species treated as a random effect.

**Parameter**	**Prevalence ratio**	**Standard error**	***P* value**
**Model fit: Conditional R^2 = 0.60, Marginal R^2 = 0.46**			
Activity	3.15	1.00	< 0.001
Cell size × Nematode [Dv]	0.22	0.11	0.004
Cell size × Nematode [Pa]	1.80	0.90	0.244
Gram × Nematode [Dv]	1.50	0.32	0.067
Gram × Nematode [Pa]	0.87	0.19	0.530
Antagonism [Low] × Nematode [Dv]	1.36	0.28	0.146
Antagonism [Low] × Nematode [Pa]	1.31	0.27	0.195
Antagonism [Moderate] × Nematode [Dv]	1.67	0.28	0.004
Antagonism [Moderate] × Nematode [Pa]	1.80	0.31	0.001
